# Synergistic function of four novel thermostable glycoside hydrolases from a long-term enriched thermophilic methanogenic digester

**DOI:** 10.3389/fmicb.2015.00509

**Published:** 2015-05-22

**Authors:** Meng Wang, Guo-Li Lai, Yong Nie, Shuang Geng, Liming Liu, Baoli Zhu, Zhongping Shi, Xiao-Lei Wu

**Affiliations:** ^1^School of Biotechnology, Jiangnan UniversityWuxi, China; ^2^Department of Energy and Resources Engineering, College of Engineering, Peking UniversityBeijing, China; ^3^Institute of Engineering (Baotou), College of Engineering, Peking UniversityBaotou, China; ^4^Institute of Microbiology, Chinese Academy of SciencesBeijing, China

**Keywords:** cellulase, xylanase, β-xylosidase, β-glucosidase, metagenome, enzyme compatibility, enzyme cocktail, biofuels production

## Abstract

In biofuel production from lignocellulose, low thermostability and product inhibition strongly restrict the enzyme activities and production process. Application of multiple thermostable glycoside hydrolases, forming an enzyme “cocktail”, can result in a synergistic action and therefore improve production efficiency and reduce operational costs. Therefore, increasing enzyme thermostabilities and compatibility are important for the biofuel industry. In this study, we reported the screening, cloning and biochemical characterization of four novel thermostable lignocellulose hydrolases from a metagenomic library of a long-term dry thermophilic methanogenic digester community, which were highly compatible with optimal conditions and specific activities. The optimal temperatures of the four enzymes, β-xylosidase, xylanase, β-glucosidase, and cellulase ranged from 60 to 75°C, and over 80% residual activities were observed after 2 h incubation at 50°C. Mixtures of these hydrolases retained high residual synergistic activities after incubation with cellulose, xylan, and steam-exploded corncob at 50°C for 72 h. In addition, about 55% dry weight of steam-exploded corncob was hydrolyzed to glucose and xylose by the synergistic action of the four enzymes at 50°C for 48 h. This work suggested that since different enzymes from a same ecosystem could be more compatible, screening enzymes from a long-term enriching community could be a favorable strategy.

## Introduction

Enzymes catalyzing the hydrolysis of cellulose and hemicellulose are a source of worldwide interest for alternative bio-energy development and other environmental applications, such as treating wastewater from the dying, textile and pulping industries. Many enzymes are used in these processes, including exoglucanase (EC 3.2.1.91), endoglucanase (EC 3.2.1.4), and β-glucosidase (EC 3.2.1.21) for cellulose hydrolysis; xylanase (EC 3.2.1.8) and β-xylosidase (EC 3.2.1.37) to hydrolyze xylan; and α-L-arabinofuranosidase (EC 3.2.1.55) and α-glucuronidase (EC 3.2.1.39) to hydrolyze side chains in hemicellulose (Beg et al., 2001). To utilize these enzymes in biofuel production, two processes are generally used: separate hydrolysis and fermentation (SHF), and simultaneous saccharification and fermentation (SSF). Temperatures of hydrolysis in SHF and SSF are around 50 and 32°C, respectively (Tomas-Pejo et al., 2008). SHF has been reported to be more efficient and cost-effective than SSF (Magnus et al., 2012) because it uses thermostable enzymes, which improves the solubility of substrates and enhances the mass transfer rate, while reducing cooling requirements and the probability of bacterial contamination (Krahe et al., 1996). However, product inhibition can be a major source of inefficiency in SHF, especially the inhibition on cellulase and xylanase (Zhang and Lynd, 2004). Therefore, combinations of relevant enzymes, forming a so-called enzyme cocktail, have been intensively investigated in recent decades (Zhou et al., 2009; Banerjee et al., 2010). For example, the combination of exoglucanase, endoglucanase, and β-glucosidase is used to increase cellulose degradation efficiency (Gusakov et al., 2007), and the combination of xylanase and β-xylosidase has been shown to enhance hemicellulose hydrolysis (Fan et al., 2009). In general, these cocktails are formed from widely used commercial enzymes, such as the cellobiohydrolases I and II and endoglucanases I and II from the fungus *Trichoderma reesei* (Henrissat et al., 1985). However, since the host microorganisms from which these enzymes were originally isolated are from different habitats and have different optimal growth conditions, the isolated enzymes generally also have different optimal conditions, and may not be compatible in the same cocktail.

Metagenomic approaches for screening novel enzymes have the potential to increase the compatibility of the candidate enzymes in a cocktail, because these metagenome-oriented enzymes can be from the same microbial community, living in the same habitat, and even in the same niche. For example, hundreds of lignocellulose hydrolases were screened from metagenomic libraries of the rumen (Hess et al., 2011), the gut system of termites (Warnecke et al., 2007), soil (Mcandrew et al., 2013), and biogas digesters (Yan et al., 2013). Nevertheless, more hydrolases are believed to be awaiting discovery in the “microbial dark matter” (Rinke et al., 2013). So far, most of this research has focused on the desirable properties of the discovered enzymes, such as thermostability, ionic strength, alkaline tolerance, activity at cold temperatures, and wide substrate specificity. Comparatively few efforts have been made to investigate their compatibility and synergistic actions (Del Pozo et al., 2012; Geng et al., 2012; Gruninger et al., 2014).

For obtaining thermostable enzymes, we constructed a metagenomic library from a dry thermophilic methanogenic digester community enriched mainly with paper for over 10 years (Tang et al., 2011). We isolated and identified four novel thermostable hydrolases and found them to have high compatibility as a synergistic cocktail for hydrolyzing cellulose, xylan, and steam-exploded corncob. This work further proved that metagenomic screening is an effective way for finding compatible enzymes.

## Materials and methods

### Construction, functional screening and *in silico* analysis of fosmid metagenomic library

The genomic DNA of the thermophilic methanogenic digester community, as described previously (Tang et al., 2011), was extracted using FastDNA® SPIN Kit for soil (MP, Santa Ana, America). The DNA purification, ligation, and transformation were performed according to the manufacturer's instructions of Copy Control™ Fosmid Library Production Kit (Epicentre, Madison, America). A fosmid library was constructed, and the analysis of insert DNA fragments was carried out as described previously (Hu et al., 2010). Activities of cellulase and xylanase were screened from the library with the Congo red method (Wood et al., 1988). To screen β-glucosidase activity, the ammonium ferric citrate-esculin hydrate method (Eberhart et al., 1964) was used, while *p*NPX (*p*-nitrophenyl-β-D-xyloside) was used to identify β-xylosidase (Poutanen and Puls, 1988). Fosmid clones with positive activities were sequenced using the GS FLX system (Roche, Basel, Switzerland). The sequences were then assembled with the putative open reading frames (ORFs) predicted using SoftBerry fgenesb (Warnecke et al., 2007). The deduced proteins were then annotated against the NCBI non-redundant database. The Pfam (Finn et al., 2014) and CAZY (Lombard et al., 2014) databases were used to identify conserved domains and glycoside hydrolase families, respectively. Signal peptides were identified using SignalP 4.1 (Petersen et al., 2011). Homology models of the proteins were predicted from the SWISS-MODEL server (Arnold et al., 2006) and visualized using PyMOL (Schrodinger, 2010). Active sites of the proteins were identified by multiple sequence alignment (Mcwilliam et al., 2013). The composition of amino acid residues was analyzed online (http://www.bio-soft.net/sms/). In total, four genes from three fosmid clones were selected for further study: *Xyl522*, coding for β-xylosidase; *Xyn526*, coding for xylanase; *Bgl8520*, coding for β-glucosidase; and *Cel1753*, coding for cellulase. Phylogenetic analysis was performed by MEGA5.1 with the best hits in BLASTP and characterized protein sequences from the same protein family, using the neighbor-joining algorithm.

### Cloning, expression, purification, and characterization of target enzymes

The four target genes were PCR amplified from the corresponding fosmids with the primers listed in Supplementary Table 1 using PrimeSTAR HS DNA Polymerase (Takara, Dalian, China) according to the manufacturer's instructions. After purification and double digestion, the PCR amplicons were cloned into pET-28a (+) (Qiagen, Hilden, Germany). Recombinants of *Xyl522, Xyn526, Bgl8520*, and *Cel1753* were further transformed into *E. coli* BL21 (DE3) (CoWin Biotech, Beijing, China). Recombinant cells were cultured in Lysogeny Broth medium with 50 μg/mL kanamycin and induced by 1 mM IPTG at 37°C for 4 h. Cells were harvested and subjected to freeze-thaw lysis. Next, the cell lysate was centrifuged at 4°C, 12,000 × *g* for 30 min, and the supernatant was collected for protein purification though Ni-NTA resin (GE Healthcare, Uppsala, Sweden) according to the manufacturer's instructions. The protein concentration was determined using the BCA Protein Assay Kit (Tiangen, Beijing, China).

Enzyme activities were detected as follows. *p*NPX (25 mM final concentration) and *p*NPG (*p*-nitrophenyl-β-D-glucoside, 25 mM final concentration) were mixed with 1 μg Xyl522 and 1 μg Bgl8520, respectively, each to a final volume of 40 μL (pH 5.0). After incubation at 50°C for 5 min, 50 μL 1 M Na_2_CO_3_ was added to terminate the reaction. The concentrations of *p*-nitrophenol product were determined by detecting the absorbance at 410 nm using spectrophotometer UV1700 (Shimadzu, Kyoto, Japan). The specific activities (U) of β-xylosidase and β-glucosidase were shown in micromole *p*-nitrophenol produced from *p*NPX and *p*NPG per minute per milligram of protein. Similarly, beech wood xylan (1%, *w/v* final concentration) and CMC (carboxymethylcellulose, 1%, *w/v* final concentration) were mixed with 5 μg Xyn526 and 5 μg Cel1753, respectively, to a final volume of 200 μL (pH 5.0). After incubation at 50°C for 15 min, the reaction mixtures were added to 500 μL dinitrosalicylic acid and boiled for 5 min to terminate the reactions (Miller, 1959). Concentrations of released xylose or glucose were determined by analyzing the absorbance of the reaction mixtures at 540 nm. Activities of cellulase and xylanase were defined as micromole xylose and glucose produced per minute per milligram of protein. The determination of filter paper activity (FPA) was performed as previously described (Zhang et al., 2010); FPA was defined as micromole glucose produced per minute per milligram of protein.

The pH and temperature optima of the four enzymes were determined in the range of pH 3.0–12.0 (Xyl522, pH 3.5–7.0; Xyn526, pH 3.5–11.0; Bgl8520, pH 4.0–9.0; and Cel1753, pH 3.5–9.0) and 20–90°C (Xyl522 and Bgl8520, 20–85°C; Xyn526, 20–90°C; and Cel1753, 20–80°C). To maintain suitable pH values, citric acid-Na_2_HPO_4_ buffer (pH 3.0–8.0), Tris-HCl (pH 8.0–10.0) and Na_2_HPO_4_-NaOH (pH 10.0–12.0) were used. For determination of optimal temperatures, pH was maintained at 5.0, for consistency with conditions in the methanogenic digester community from which the enzymes originate. To test for pH stability, the four enzymes were incubated in the buffer solutions mentioned above with corresponding pH values at 4°C for 24 h, after which their residual enzyme activities were measured as described above. For example, Xyn526 was incubated at 4°C in buffer solutions ranging from pH 3.5 to 12.0 for 24 h, after which mixtures containing 5 μg xylanase protein were used for activity determination as described above. To test the thermostability of the four enzymes, Xyl522 and Bgl8520 were incubated at 50, 55, 60, and 75°C; Xyn526 incubated at 50, 65, 70, and 75°C; and Cel1753 incubated at 50, 60, 65, and 70°C for 2 h in buffer solutions of pH 4.5, 6.0, 5.0, and 5.0, respectively. The residual activities of all enzymes were tested at 50°C and pH 5.0. Briefly, mixtures containing 1 μg Xyl522 and Bgl8520 each, or 5 μg Xyn526 and Cel1753 each were sampled every 20 min until 120 min, and enzyme activity was determined for samples taken at each time point. In addition, CMC, microcrystalline cellulose (MIC), beech wood xylan, *p*NPG, *p*NPC (*pNP*-β-D-cellobioside), pNPX, and *p*NPA (*p*NP-α-L-arabinofuranoside) were used to investigate the substrate specificity of the enzymes. *K_m_* and *V_max_* were calculated by Lineweaver–Burk plot based on enzyme activities at their optimal conditions with different substrate concentrations (1–50 mM for *p*NPG, *p*NPX, *p*NPA, and *p*NPC; 1–50 g/L for xylan beech wood, xylan oat spelt, CMC, and MIC).

### Synergistic action of enzymes on CMC, xylan, and steam-exploded corncob

The activities of Cel1753 and Bgl8520 cocktail against CMC (1%, *w/v*) and Xyn526 and Xyl522 cocktail against xylan (1%, *w/v*) were assayed by detecting the products using thin-layer chromatography (TLC). After the mixtures were incubated at 50°C, pH 5.0 for 4 h, respectively, 5 μL of each culture was spotted on TLC Silica gel 60 (Merck, Darmstadt, Germany) (Murashima et al., 2003). TLC plates were developed with ethyl acetate/methanol/acetic acid/water (12:3:3:1, *v/v*) as the mobile phase, and dyed with chromogenic agent containing 4 g diphenylamine, 4 mL phenylamine, 20 mL 85% phosphoric acid, and 200 mL acetone for 15 s followed by heating at 80°C for 10 min.

Steam-exploded corncob was prepared according to a previously described protocol (Teng et al., 2010) with a dry weight of 32%, which was composed of cellulose (~35%), hemicellulose (~38%) and lignin (~18%). The corncob biomass (2%, *w/v*) were incubated with Cel1753 (40 FPA/g, dry weight, as follows), Xyn526 (200 U/g), a cocktail of Cel1753 (40 FPA/g), and Bgl8520 (50 U/g), a cocktail of Xyn526 (200 U/g) and Xyl522 (50 U/g), and a cocktail of all four enzymes at 50°C, pH 5.0 for 72 h. Enzyme dosages used above were the minimum ones to achieve the saturated hydrolysis of corncobs by itself. The concentrations of glucose, cellobiose, xylose, and xylobiose were measured using a Prominence Liquid Chromatograph-20A (Shimadzu) according to the manufacturer's instructions. Briefly, a fluorometric detector and a shim-pack ISA-07 column were used. The system was operated at 65°C and a flow rate of 0.6 mL/min using H_3_BO_3_-KOH as the mobile phase with 1% *w/v* L-arginine for product derivatization. Calculation of saccharification efficiency was performed as described previously (Del Pozo et al., 2012).

### Nucleotide sequence accession numbers

The GenBank accession numbers of Xyl522, Xyn526, Bgl8520, and Cel1753 genes were KM982177, KM982178, KM982179, and KM982180, respectively.

## Results

### Screening of the metagenomic library and sequence analysis

The fosmid metagenomic library contained about 92,000 clones. The average length of the insert DNA fragments was 36.0 kb. Altogether, we identified 49, 37, and 92 clones harboring cellulase, xylanase and β-glucosidase activities from 9,700 clones, respectively. According to the average insert size of fosmid clones, the hit rates (gene/Mbp) of cellulase, xylanase and β-glucosidase were 1/8.1, 1/10.8, and 1/4.4, respectively, which were higher than those metagenomes from a 1.5 year enriched biogas digester (Yan et al., 2013), from buffalo rumen (Duan et al., 2009) and from soil (Kim et al., 2008), suggesting that long-term enrichment could increase the possibility of identifying the target genes. Three positive fosmids, designated F52, F85, and F175, were found positive for xylanase, β-glucosidase, and cellulase activities, respectively. Complete sequence analysis revealed that fosmids F52, F85, and F175 had inserts of 38,176, 34,045, and 37,655 bp, and contained 26, 29, and 33 ORFs, respectively (Supplementary Figures 1–3). From the inserted DNA fragments in fosmids F52, F85, and F175, putative genes coding for β-xylosidase, xylanase, β-glucosidase, and cellulase and their glycoside hydrolase (GH) family were annotated (Supplementary Table 2) and designated as *Xyl522, Xyn526, Bgl8520*, and *Cel1753*, respectively. The four putative enzymes shared the highest amino acid sequence similarities with β-xylosidase from *Clostridium stercorarium* (63%, Genbank accession WP_015357972.1), 1,4-β-xylanase from *Caldicoprobacter oshimai* (56%, Genbank accession WP_025746830.1), β-glucosidase from bacterium UASB270 (60%, Genbank accession WP_021653982.1), and cellulase from an uncultured bacterium (59%, Genbank accession AEV59736.1). Xyl522 and Bgl8520 did not have signal peptides, while Xyn526 and Cel1753 had, suggesting that Xyl522 and Bgl8520 were intracellular, while Xyn526 and Cel1753 were functional only when secreted. It is notable that the alanine, proline, arginine, and glutamic acid residue contents in our proteins were generally around 1–4% higher than those of the mesothermal enzymes, whereas the glycine, serine, lysine and aspartic acid content was around 1–4% lower (Supplementary Table 4) (Morana et al., 2007; Alvarez et al., 2013; Han et al., 2013; Mcandrew et al., 2013). These differences in relative content of specific amino acid residues could be related to the temperature adaptabilities of these enzymes.

The predicted models of enzyme structure (Supplementary Figure 4) suggested that the four enzymes all had a classical (α/β)_8_ TIM barrel fold structure. Xyl522, a member of the GH 3 family, also contained an immunoglobulin-like region, which has been proven to contribute to both substrate binding and dimerization in this family (Mcandrew et al., 2013). Additionally, the distance between the two active sites in Xyl522 was calculated as 5.4 Å, In Bgxa1, a multifunctional β-glucosidase/β-xylosidase/α-arabinosidase, the same distance is 6.1 Å (Gruninger et al., 2014). Although Bgxa1 and Xyl522 are from the same GH 3 family, this distinction might suggest different substrate specificities.

### Cloning, expression, purification, and characterization of Xyl522, Xyn526, Bgl8520, and Cel1753

Xyl522, Xyn526, Bgl8520, and Cel1753, without their signal peptides, were heterologously expressed and purified. Plate-based function screening confirmed each protein was responsible for the assumed activities, further supporting the results of sequence alignment. Xyn526 and Cel1753 showed 79.6 U/mg and 174.0 U/mg specific activities for the hydrolysis of beech wood xylan and CMC, respectively. However, Xyn526 had relatively weak hydrolyzing activity against *p*NPX, *p*NPG, and CMC (Table 1). Cel1753 also exhibited hydrolyzing activity for MIC and filter paper, suggesting that it has potential for hydrolyzing cellulose alone. These results revealed that Cel1753 harbored a high proportion of exoglucanase activity relative to its endoglucanase activity (21.8%). This is in contrast to most cellulases, which do not possess both activities (Kim et al., 2008; Ko et al., 2011). Xyl522 could hydrolyze *p*NPX more effectively than *p*NPA or *p*NPG. Bgl8520 could hydrolyze *p*NPG 50 times more efficiently than *p*NPC (Table 2), which combined with its relatively low *K_m_* for *p*NPG indicated a higher affinity and efficiency for glycosidic bonds in cellobiose. In addition, Bgl8520 has a higher affinity toward *p*NPC than other β-glucosidases whose *K*_m_ are normally between 4.8 and 17.6 mM (Del Pozo et al., 2012), indicating its strong capacity on cellotriose hydrolysis. It is notable that no obvious activities of Xyl522 and Bgl8520 were detected against CMC, MIC, or xylan. Our results therefore suggest that *p*NPG, xylan from beech wood, *p*NPX, and CMC were the optimal substrates for Xyl522, Xyn526, Bgl8520, and Cel1753, respectively.

**Table 1:** **Substrate specificity of Xyl522, Xyn526, Bgl8520, and Cel1753**.

	**Relative activities (%)**			
	**Xyl522**	**Xyn526**	**Bgl8520**	**Cel1753**
Xylan	9.3 ± 1.4^a^	100.0 ± 3.1	ND^b^	3.1 ± 1.2
CMC	ND	2.3 ± 1.0	4.4 ± 0.7	100.0 ± 2.3
*p*NPX	100.0 ± 1.3	53.2 ± 2.5	3.2 ± 1.5	ND
*p*NPG	7.1 ± 2.7	13.1 ± 1.2	100.0 ± 2.8	33.2 ± 3.3
*p*NPA	26.5 ± 3.1	4.2 ± 1.3	ND	ND
MIC	ND	ND	ND	21.8 ± 4.1
Filter paper	ND	ND	ND	7.6 ± 2.2

a *Standard deviations are shown from triplicate measurements*.

b *No activity detected*.

**Table 2:** **Kinetic parameters of Xyl522, Xyn526, Bgl8520, and Cel1753**.

**Enzyme**	**Substrate**	***K_m_*(mM)**	***k_cat_*(s^−1^)**	***k_cat_*/*K_m_*(s^−1^· M^−1^)**
Bgl8520	*p*NPG	0.9	124.0	1.4 × 10^5^
	*p*NPX	0.7	0.3	4.3 × 10^2^
	*p*NPC	2.7	8.2	3.0 × 10^3^
Xyl522	*p*NPG	15.7	1.4	89.2
	*p*NPX	8.8	14.3	1.6 × 10^3^
	*p*NPA	10.0	3.2	3.2 × 10^2^
		***K_m_*(g· L^−1^)**	***k_cat_*(s^−1^)**	***k_cat_*/*K_m_*(L· g^−1^· s^−1^)**
Xyn526	Xylan beechwood	6.3	91.1	14.5
	Xylan oat spelt	10.2	70.5	6.9
Cel1753	CMC	8.3	70.6	8.5
	MIC	19.5	37.2	1.9

**Figure 1:**
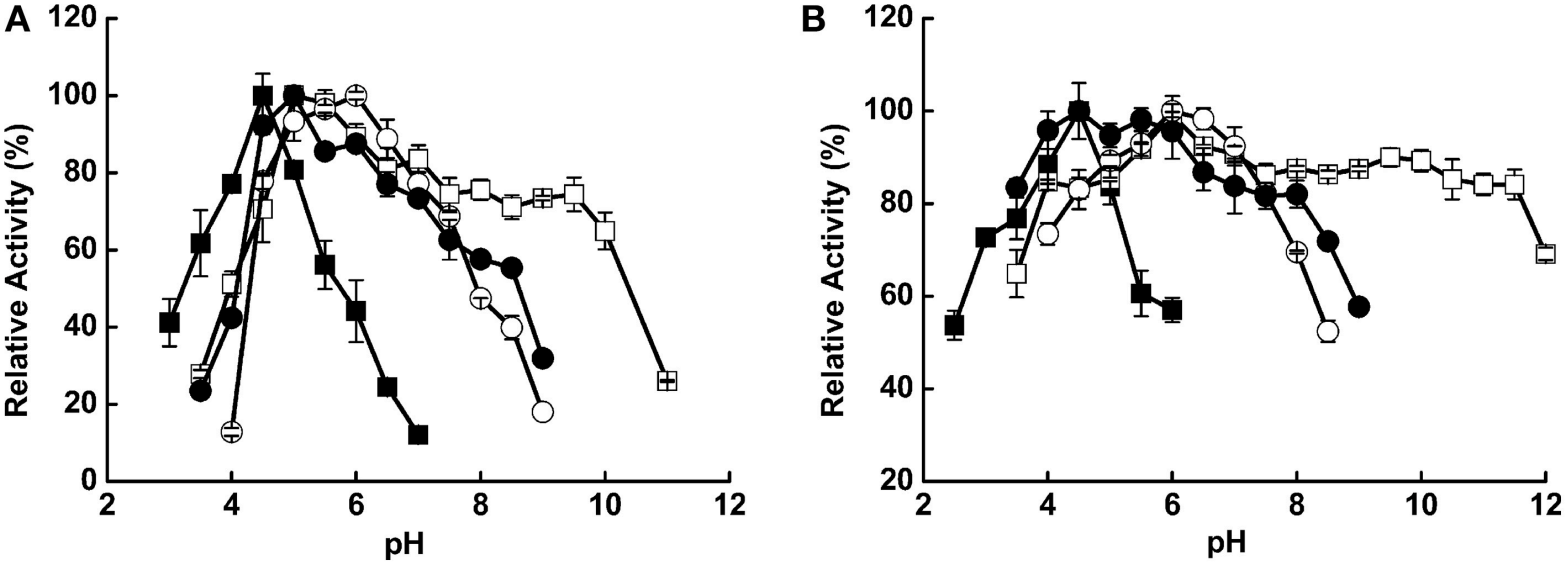
**pH optima (A) and stability (B) of Xyl522, Xyn526, Bgl8520, and Cel1753**. Activities of Xyl522, Xyn526, Bgl8520, and Cel1753 are shown as solid square, open square, open circle, and solid circle, respectively. Enzymes activities were determined with *p*NPX, xylan, *p*NPG, and CMC as substrates for Xyl522 (pH 3.5–7.0), Xyn526 (pH 3.5–11.0), Bgl8520 (pH 4.0–9.0), and Cel1753 (pH 3.5–9.0), respectively. Standard deviations are shown from triplicate measurements.

The pH value influenced hydrolysis activity and enzyme stability differently among the four enzymes. The optimal pH values were 4.5, 5.0, 6.0, and 5.0 for Xyl522, Xyn526, Bgl8520, and Cel1753, respectively (Figure 1A). Xyl522 had a narrow pH range, with over 80% hydrolysis activity between pH 4.0 and 5.0, and dramatically lower activity at pH values outside this range. In contrast, Xyn526, Cel1753, and Bgl8520 kept over 80% hydrolysis activity within the pH ranges 4.0–11.0, 3.5–8.0, and 4.5–7.5, respectively. Xyn526 and Cel1753 had high pH stabilities, retaining greater than 80% enzyme activity after incubation at 4°C for 24 h in pH 4.0–12.0 and pH 3.5–8.0, respectively (Figure 1B).

**Figure 2:**
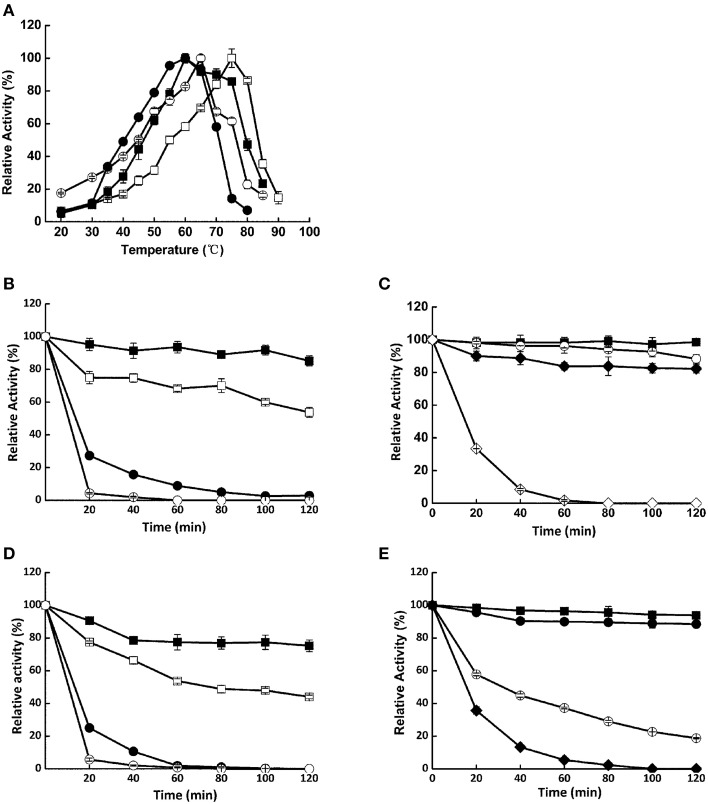
**Temperature optima (A) and stability (B–E) of Xyl522, Xyn526, Bgl8520, and Cel1753. (A)** Activities of Xyl522, Xyn526, Bgl8520, and Cel1753 are shown as solid square, open square, open circle, and solid circle, respectively. **(B–E)** Activities of enzymes at 50, 55, 60, 65, 70, and 75°C are shown as solid square, open square, solid circle, open circle, solid diamond, and open diamond, respectively. Enzymes activities were determined with *p*NPX, xylan, *p*NPG, and CMC as substrates for Xyl522, Xyn526, Bgl8520, and Cel1753, respectively. Standard deviations are shown from triplicate measurements.

Xyn526 had the highest optimal temperature of 75°C (Figure 2A); the corresponding values for Xyl522, Bgl8520, and Cel1753 were 60, 65, and 60°C, respectively. These optimal temperatures were generally higher than lignocellulose hydrolases isolated from other metagenomic libraries so far, the majority of which were below 60°C (Duan and Feng, 2010; Yan et al., 2013). In addition, Xyl522, Xyn526, Bgl8520, and Cel1753 had notably high temperature stabilities, retaining over 80% residual activity after incubation at 50°C for 2 h (Figure 2), with thermal half-life being 14, 48, 10, and 32 h, respectively (Supplementary Table 3). Even after 2 h incubation at 70°C, Xyn526 still had 82.3% residual activity toward xylan, whose thermal half-life was impressively 8 h under the same condition. In comparison, the thermostabilities of many other metagenomic enzymes are relatively weak. For example, cellulase En1 from a 40°C biogas digester metagenome (Yan et al., 2013) and glucoside hydrolase Bgxa1 from a dairy cow rumen metagenome (Gruninger et al., 2014) could only retain 80 and 10% activities respectively after incubation at 50°C for 1 h. Xylanase Xyn10N18 from a dairy cow rumen metagenome (Gong et al., 2013) and β-xylosidase RuBG3B from a yak rumen metagenome (Bao et al., 2012) lost all of their activity under the same conditions. Similarly, the alanine, proline, arginine, and glutamic acid content in these proteins were also generally less than those of our four enzymes, while the glycine, serine, lysine, and aspartic acid content was higher. For instance, the percentages of alanine, proline, and arginine residues in cellulase En1 were 4, 2, and 2% less respectively than those in Cel1753; glutamic acid content was the same between the two enzymes. In contrast, the percentages of aspartic acid and lysine residuals in cellulase En1 were 1 and 3% higher respectively than those in Cel1753, although they shared the same percentage of glycine and serine content.

### Synergistic hydrolysis of CMC, xylan, and steam-exploded corncob

**Figure 3:**
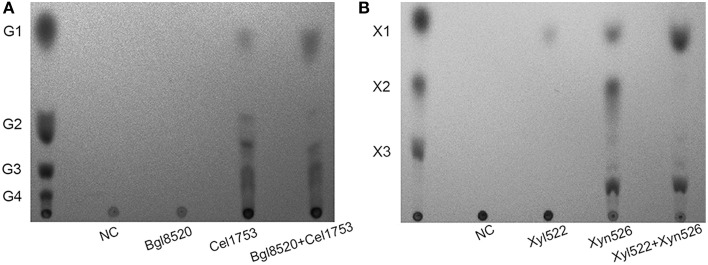
**TLC spots of the four enzymes against CMC (A) and beech wood xylan (B)**. The enzymes were incubated with substrate at 50°C, pH 5.0 for 4 h. The products were developed by TLC and stained. G1, G2, G3, and G4 are glucose, cellobiose, cellotriose, and cellotetraose, respectively. X1, X2, and X3 are xylose, xylobiose, and xylotriose, respectively. NC: negative control without addition of enzymes.

Cel1753 could hydrolyze CMC to produce mainly cello-oligosaccharides along with small amounts of glucose and cellobiose, whereas the Bgl8520 could hardly hydrolyze CMC (Figure 3A). When a cocktail of Bgl8520 and Cel1753 was added to CMC, production of glucose increased markedly compared with Cel1753 working alone, whereas the production of cellobiose and other cello-oligosaccharides decreased, suggesting the efficient degradation of CMC. Similarly, the cocktail of Xyn526 and Xyl522 resulted in higher xylose production from beech wood xylan, with xylobiose completely hydrolyzed (Figure 3B).

**Figure 4:**
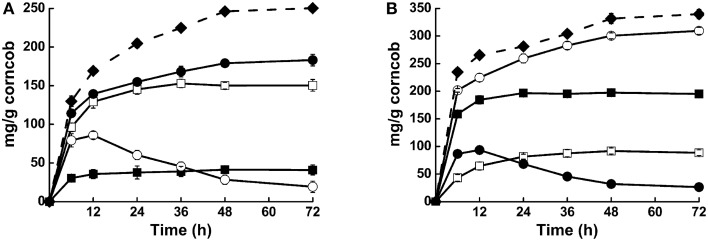
**Activities of different enzyme cocktails against steam-exploded corncob. (A)** Hydrolysis capacities of Cel1753 (open square: cellubiose production; solid square: glucose production), combination of Bgl8520 and Cel1753 (open circle: cellubiose production; solid circle: glucose production), and the four-enzyme cocktail on steam-exploded corncob (solid diamond: glucose production). **(B)** Hydrolysis capacities of Xyn526 (solid square: xylobiose production; open square: xylose production), combination of Xyl522 and Xyn526 (solid circle: xylobiose production; open circle: xylose production), and the four-enzyme cocktail on steam-exploded corncob (solid diamond: xylose production). Standard deviations are shown from triplicate measurements.

Synergistic action of these enzymes was also detected when steam-exploded corncob was used as substrate. Compared with Cel1753 alone, addition of Bgl8520 significantly increased the yield of glucose from steam-exploded corncob from 11.8 to 52.3%, while the yield of cellobiose in this cocktail decreased dramatically (Figure 4A). Moreover, glucose production was much quicker in the first 12 h, revealing the synergistic action of the two enzymes and its benefit. Similarly, the supplementation of Xyn526 with Xyl522 resulted in a 56.2% increase in the yield of xylose. This supplementation also simultaneously accelerated the hydrolysis of xylan by consuming xylobiose, which might be attributed to a reduction of Xyn526 inhibition (Figure 4B). When all four enzymes were pooled in a cocktail, xylose and glucose production increased by around 8.3 and 20.5%, respectively, resulting in total hydrolysis efficiency for cellulose and hemicellulose of 71.5 and 87.1% in 48 h at 50°C, respectively (Figure 4). This incremental effect on catalytic efficiency was higher than seen in previous studies of metagenomic enzymes. For example, supplying a metagenomic β-glucosidase into commercial *Trichoderma reesei* cellulase resulted in a relative increase of 20% glucose yield from corn stover at 50°C (Del Pozo et al., 2012). The mixture of two metagenomic enzymes, β-xylosidase and xylanase, resulted in a relative increase of 37% yield at 40°C from beech wood xylan compared with xylanase alone (Gruninger et al., 2014).

## Discussion

In the biofuel industry, increasing the thermostability of enzymes is important for reducing operational cost. Many attempts have been made to isolate thermostable enzymes, from organisms such as *Clostridium absonum* (Rani and Nand, 2000) and *Thermoascus aurantiacus* (Hong et al., 2007). Because of product inhibition, using of a single enzyme usually leads to low efficiency in the lignocellulose hydrolysis process (Gusakov and Sinitsyn, 1992). However, enzyme cocktails composed of different enzymes can form a “working team”, which is similar as the different enzymes in a single cell (Nie et al., 2014), to sequentially degrade lignocellulose and its intermediates. These enzyme cocktails have proven to be a suitable solution to product inhibition and have therefore drawn worldwide research interest (Chundawat et al., 2011; Gao et al., 2011). To form an efficient cocktail, candidate enzymes need to have compatible pH and temperature optima as well as compatible specific activities. Cocktails have generally been based on commercial enzymes isolated from different microorganisms, which may have different optimal growth conditions. Recently, the isolation of novel enzymes, including thermostable enzymes from metagenomic libraries, has been extensively studied, leading to the isolation of many enzymes with special characteristics such as being cold- (Wierzbicka-Wos et al., 2013) or heat-active (Schroder et al., 2014), and ionic liquid- (Ilmberger et al., 2012) or product-tolerant (Lu et al., 2013). Microorganisms in the same metagenome generally live together for a relatively long time, and should have similar tolerance for environmental conditions, such as temperature, pH, and oxidation-reduction potentials, which may result in isolation of enzymes with similar optimal conditions. Metagenomic screening may therefore offer a way of increasing the compatibility of different candidate enzymes in a cocktail. However, to date there have been few reports of successful trials using a cocktail of enzymes from the same metagenome. Therefore, we identified four novel thermostable enzymes and reported the first trial of a combination of compatible enzymes from the same metagenome.

The four enzymes we isolated from a long-term enriched thermophilic microbial community had higher thermostability (Figure 2) than those from metagenomes sampled from mesophilic soil, sea sediment, and animal gastrointestinal tracts, whose optimal temperatures were generally under 60°C (Duan and Feng, 2010), and whose thermostabilities were relatively poor (Feng et al., 2007; Gong et al., 2012). Although a β-glucosidase has been previously found (from screening a hydrothermal spring metagenome) with an optimal temperature of 90°C (Schroder et al., 2014), its observed relative activity at 50°C was only 20%. Similarly, although one β-glucosidase (LAB25g2 from cow rumen) retains as much as 82% activity after 5 days of incubation at 50°C, it required several days to consume cellobiose completely, suggesting its high thermostability with relatively low hydrolysis efficiency (Del Pozo et al., 2012). We attribute the high thermostabilities of our four enzymes to the fact that they were isolated from a thermophilic methanogenic digester community. This community has been enriched with paper at 53°C for longer than 10 years (Tang et al., 2011), which should have provided a stronger high-temperature selective pressure than that found in the gastrointestinal tracts of ruminants (Yan et al., 2013). Furthermore, the unusual amino acid residue composition of these enzymes may also contribute to their thermostabilities. Lu and colleagues, 1998 reported that there are remarkable differences in amino acids composition between mesophilic and thermophilic proteins. In this research, although the thermostable enzymes we isolated share high amino acid sequence similarities with the mesothermal stable enzymes (Supplementary Figures 5–8), the thermostable enzymes have higher relative ratios of alanine, proline, arginine, and glutamic acid residues and lower ratios of glycine, serine, lysine, and aspartic acid residues (Supplementary Table 4). Alanine tends to form a stable helix, while the reduced number of glycine residues could lessen flexibility of loops and therefore provide heat resistance (Yip et al., 1995). The structural entropy of proline is less than other amino acids as it requires more energy to unfold; its presence can therefore enhance the thermostability of a protein (Vogt et al., 1997). The presence of arginine, which has a large side chain with a delocalized positive charge, can promote the formation of salt bridges and hydrogen bonds to enhance protein stability (Kumar et al., 2000). Lower levels of hydrophilic amino acids such as serine and lysine have also been linked with thermostability (Argos et al., 1979; Lu et al., 1998). Therefore, we propose that the unusual amino acid composition of the four enzymes we isolated did not change their molecular conformation, but did increase their thermostability (Figure 2).

Using a cocktail composed of the four lignocellulose hydrolases, we observed enhanced hydrolysis of steam-exploded corncob after incubation at 50°C for 72 h, suggesting the compatible thermostabilities and synergistic cooperation of these enzymes. It is reasonable that Xyl522 and Bgl8520 could prevent product inhibition of Xyn526 and Cel1753, respectively. As cellulose is often embedded in hemicellulose and lignin (Jeoh et al., 2007), the hydrolytic activity of Xyn526 on hemicellulose could expose more cellulose to Cel1753, along with Xyl522 and Bgl8520 in the four-enzyme cocktail, which could further improve the yield of monosaccharide and stimulate lignocellulose hydrolysis in this community (Figure 4). Indeed, Xyl522 and Xyn526 were cloned from the same fosmid clone (F52), suggesting they might be from the same host organism and potentially hydrolyze xylan synergistically *in situ*. The presence of a signal peptide in Xyn526 indicates that it is secreted out of cells to hydrolyze xylan. The resulting oligosaccharides, such as xylo-oligosaccharides and arabino-oligosaccharides, could be then transported into the cytoplasm by carbohydrate ABC transporters, which were found to be enriched in F52. Glucoside hydrolases including Xyl522 could then degrade these oligosaccharides into monosaccharides like xylose and arabinose for further metabolism (Supplementary Figure 1). In addition, the extracellular Cel1753 could hydrolyze cellulose outside cells, which might also be transported into the cells with enriched ABC transporters, such as those found in the F85 fosmid clone (Supplementary Figure 2). These could then be hydrolyzed by non-signal peptide β-glucosidases, such as Bgl8520. It is reasonable to assume that such a complex cooperation can only be achieved in a cocktail of enzymes from the same well-enriched metagenome, because all the derived enzymes had similar optimal temperatures, thermostabilities, and pH values, as well as compatible specific activities, and this favorable compatibility was the basis of their synergism and high efficiency. It is notable that our enzyme cocktail was relatively weak in hydrolysis capacity compared with cocktail Accellerase®Trio™, which is composed of cellulase, xylanase, and β-glucosidase, and can hydrolyze 75–90% of glucan and xylan at 50°C in 72 h. In order to improve the catalytic efficiency of our cocktail to reach industrial standards, we also need to use the strategy of developing industrial enzymes, such as molecular modification (Banerjee et al., 2010). In addition, since the original thermophilic methanogenic digester community could nearly completely convert paper to methane within 72 h (Tang et al., 2011), there should be more powerful enzymes, for more powerful enzyme cocktail, still in the dark matter waiting to be explored.

In summary, four novel thermostable glycoside hydrolases were isolated and identified from a metagenomic library constructed from a long-term dry thermophilic methanogenic digester community. The isolated enzymes and their mixed cocktail were highly effective in the hydrolysis of lignocellulose such as steam-exploded corncob. Since enzymes from the same metagenome may have high compatibilities and act synergistically, these experiments offer a way of screening compatible enzymes to form highly efficient enzyme cocktails.

## Author contributions

MW, YN, ZS, and XW designed and coordinated the study and write the manuscript. MW, GS, GL, and BZ carried out the experiments and analyzed the results. LL and XW contributed preparing the manuscript. All authors read and approved the final manuscript. ZS, YN, and XW contributed equally to this work.

### Conflict of interest statement

The authors declare that the research was conducted in the absence of any commercial or financial relationships that could be construed as a potential conflict of interest.

## Abbreviations

SHF: separate hydrolysis and fermentation
SSF: simultaneous saccharification and fermentation
GH: glycoside hydrolase
CMC: carboxymethylcellulose
*p*NPX: *p*-nitrophenyl-β-D-xyloside
*p*NPG: *p*-nitrophenyl-β-D-glucoside
MIC: microcrystalline cellulose
*p*NPA: *p*NP-α-L-arabinofuranoside
*p*NPC: *pNP*-β-D-cellobioside
ORF: open reading frame
FPA: filter paper activity
TLC: thin-layer chromatography.
