# Supplementary material for: Synergistic function of four novel thermostable glycoside hydrolases from a long-term enriched thermophilic methanogenic digester
Source: Front Microbiol. 2015 May 22;6:509. doi: 10.3389/fmicb.2015.00509 (PMC4441150; doi:10.3389/fmicb.2015.00509)
Supplement: Supplementary file 1 [file Table1.DOCX]

**Table 1. Oligonucleotides primers used in this work**

|  | Primers (5’-3’)^a^ | Restriction sites |
| --- | --- | --- |
| Xyl522F | GGAATTCCATATGCTTTTCCAGCGAAGTGACCTGC | *Nde* I |
| Xyl522R | CCCAAGCTTTCAGCGGGGCAGCTCCAG | *Hind* III |
| Xyn526F | GGAATTCCATATGGAAGCTGGAGGTGTTCCCAT | *Nde* I |
| Xyn526R | CCCAAGCTTTTAGCGATCCTGTGCAGTGCT | *Hind* III |
| Bgl8520F | CATGCCATGGAGATACAAATGAATAAACAT | *Nco* I |
| Bgl8520R | CCCAAGCTTGGCATAAAGGGCCTCC | *Hind* III |
| Cel1753F | CATGCCATGGCGAAAACCGGCGATCAG | *Nco* I |
| Cel1753R | CCCAAGCTTCTGGGCATATCTGATCATCG | *Hind* III |

a: Restriction enzyme sites are underlined.
