# Supplementary material for: Synergistic function of four novel thermostable glycoside hydrolases from a long-term enriched thermophilic methanogenic digester
Source: Front Microbiol. 2015 May 22;6:509. doi: 10.3389/fmicb.2015.00509 (PMC4441150; doi:10.3389/fmicb.2015.00509)
Supplement: Supplementary file 2 [file Table2.DOCX]

**Table 2. *In silico* analysis of lignocellulose hydrolases in fosmid clones F52, F85 and F175**

|  | Best hit | Accession | Sequence Identities | Putative Mol. wt. (kDa) | Protein Domains |
| --- | --- | --- | --- | --- | --- |
| F52-1 | α-Glucuronidase | WP_011887512.1 | 45% | 28.1 | Glyco_hydro_67_N, |
|  |  |  |  |  | Glyco_hydro_67_M |
| F52-2  (Xyl522) | β-Xylosidase | WP_015357972.1 | 63% | 78.9 | Glyco_hydro_3_C, |
|  |  |  |  |  | Glyco_hydro_3_N, |
|  |  |  |  |  | Fn3-like |
| F52-6  (Xyn526) | 1,4-β-Xylanase | WP_025746830.1 | 56% | 43.4 | Glyco_hydro_10 |
| F52-14 | Glycoside hydrolase | WP_026298195.1 | 58% | 132.2 | Glyco_hydro_2_N, |
|  |  |  |  |  | Glyco_hydro_2, |
|  |  |  |  |  | Glyco_hydro_2_C |
| F52-18 | Hypothetical protein | WP_019640267.1 | 49% | 68.9 | Glyco_hydro_2_N |
| F85-20  (Bgl8520) | β-Glucosidase | WP_021653982.1 | 59% | 53.2 | Glyco_hydro_1 |
| F175-3  (Cel1753) | Putative cellulase | AEV59736.1 | 59% | 51.7 | Glyco_hydro_5 |
