# Supplementary material for: Synergistic function of four novel thermostable glycoside hydrolases from a long-term enriched thermophilic methanogenic digester
Source: Front Microbiol. 2015 May 22;6:509. doi: 10.3389/fmicb.2015.00509 (PMC4441150; doi:10.3389/fmicb.2015.00509)
Supplement: Supplementary file 3 [file Table3.DOCX]

**Table 3 Thermal half-life (hour) of Xyl522, Xyn526, Bgl8520, and Cel1753**

|  | Xyl522 | Xyn526 | Bgl8520 | Cel1753 |
| --- | --- | --- | --- | --- |
| 50°C | 14 | 48 | 10 | 32 |
| 55°C | 3 | 45 | 1.7 | 23 |
| 60°C | 0.4 | 37 | 0.3 | 11 |
| 65°C | 0.2 | 25 | 0.2 | 0.6 |
| 70°C | --^a^ | 8 | -- | 0.4 |
| 75°C | -- | 0.3 | -- | -- |

a: Half-life was too short to identify.
