# Supplementary material for: Synergistic function of four novel thermostable glycoside hydrolases from a long-term enriched thermophilic methanogenic digester
Source: Front Microbiol. 2015 May 22;6:509. doi: 10.3389/fmicb.2015.00509 (PMC4441150; doi:10.3389/fmicb.2015.00509)
Supplement: Supplementary file 4 [file Table4.DOCX]

**Table 4. Overview of amino acid residue compositions of Xyl522, Xyn526, Bgl8520, and Cel1753**

|  | Xyl522 | | Xyn526 | | Bgl8520 | | Cel1753 | |
| --- | --- | --- | --- | --- | --- | --- | --- | --- |
| Pattern | Times Found | Percentage | Times Found | Percentage | Times Found | Percentage | Times Found | Percentage |
| A | 77 | 11 | 33 | 9 | 31 | 7 | 40 | 9 |
| C | 13 | 2 | 0 | 0 | 3 | 1 | 4 | 1 |
| D | 31 | 4 | 28 | 7 | 32 | 7 | 38 | 8 |
| E | 65 | 9 | 30 | 8 | 36 | 8 | 36 | 8 |
| F | 23 | 3 | 15 | 4 | 19 | 4 | 17 | 4 |
| G | 61 | 8 | 26 | 7 | 38 | 8 | 27 | 6 |
| H | 18 | 3 | 11 | 3 | 9 | 2 | 10 | 2 |
| I | 28 | 4 | 17 | 4 | 31 | 7 | 27 | 6 |
| K | 26 | 4 | 18 | 5 | 31 | 7 | 19 | 4 |
| L | 82 | 11 | 34 | 9 | 35 | 8 | 26 | 6 |
| M | 10 | 1 | 6 | 2 | 6 | 1 | 11 | 2 |
| N | 24 | 3 | 15 | 4 | 20 | 4 | 26 | 6 |
| P | 43 | 6 | 21 | 5 | 21 | 5 | 21 | 5 |
| Q | 26 | 4 | 12 | 3 | 16 | 4 | 16 | 4 |
| R | 44 | 6 | 27 | 7 | 20 | 4 | 27 | 6 |
| S | 35 | 5 | 14 | 4 | 17 | 4 | 22 | 5 |
| T | 32 | 4 | 12 | 3 | 13 | 3 | 24 | 5 |
| V | 48 | 7 | 32 | 8 | 30 | 7 | 34 | 7 |
| W | 8 | 1 | 15 | 4 | 16 | 4 | 9 | 2 |
| Y | 25 | 3 | 19 | 5 | 28 | 6 | 21 | 5 |
| Aliphatic I,L,V | 158 | 22 | 83 | 22 | 96 | 21 | 87 | 19 |
| Aromatics F,W,Y | 56 | 8 | 49 | 13 | 63 | 14 | 47 | 10 |
| Positive K,R,H | 88 | 12 | 56 | 15 | 60 | 13 | 56 | 12 |
| Negative D,E | 96 | 13 | 58 | 15 | 68 | 15 | 74 | 16 |
