# Supplementary material for: Synergistic function of four novel thermostable glycoside hydrolases from a long-term enriched thermophilic methanogenic digester
Source: Front Microbiol. 2015 May 22;6:509. doi: 10.3389/fmicb.2015.00509 (PMC4441150; doi:10.3389/fmicb.2015.00509)
Supplement: Supplementary file 7 [file Image3.PDF]

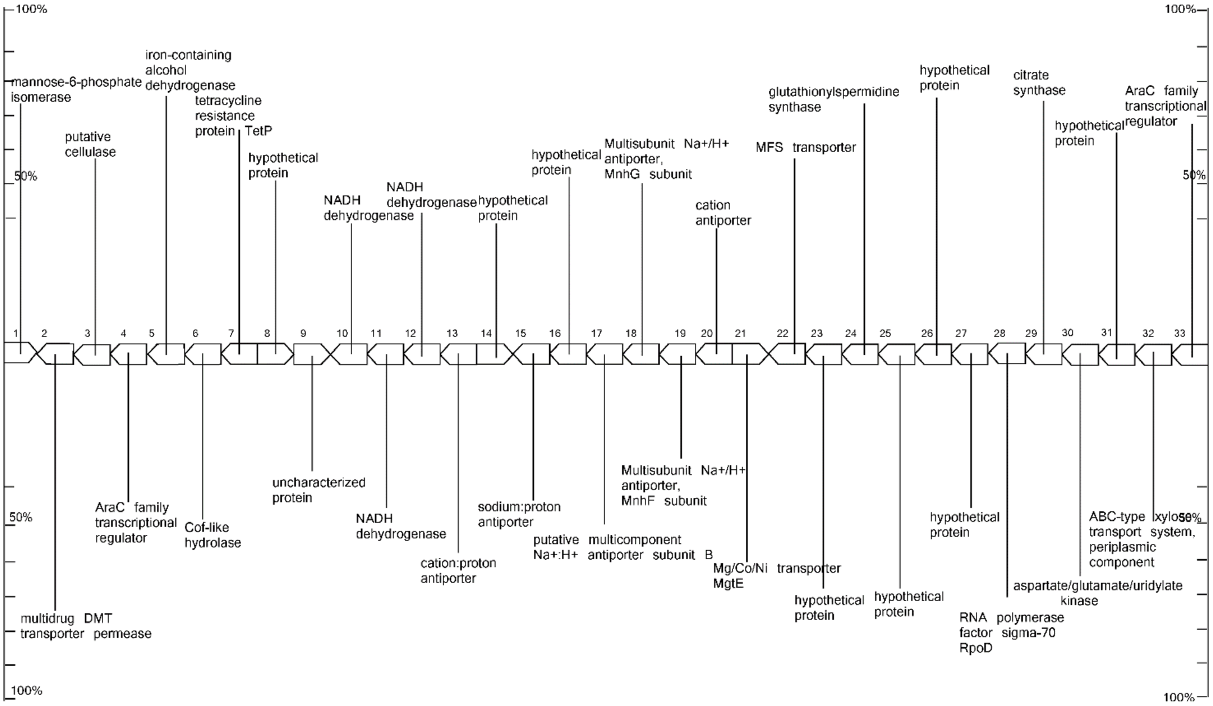

Supplementary Figure 3. Distribution of open reading frames in fosmid clones F175.

The sequences were submit to softberry for ORF prediction, and the annotation was performed by BLAST against non-redundant protein sequences database and Pfam A database. The transcription direction is represented by arrows, the Y-axis represents the identities of ORF with corresponding annotation hit. The length of ORF and noncoding region were uncharted.
