# Supplementary material for: Synergistic function of four novel thermostable glycoside hydrolases from a long-term enriched thermophilic methanogenic digester
Source: Front Microbiol. 2015 May 22;6:509. doi: 10.3389/fmicb.2015.00509 (PMC4441150; doi:10.3389/fmicb.2015.00509)
Supplement: Supplementary file 8 [file Image4.PDF]

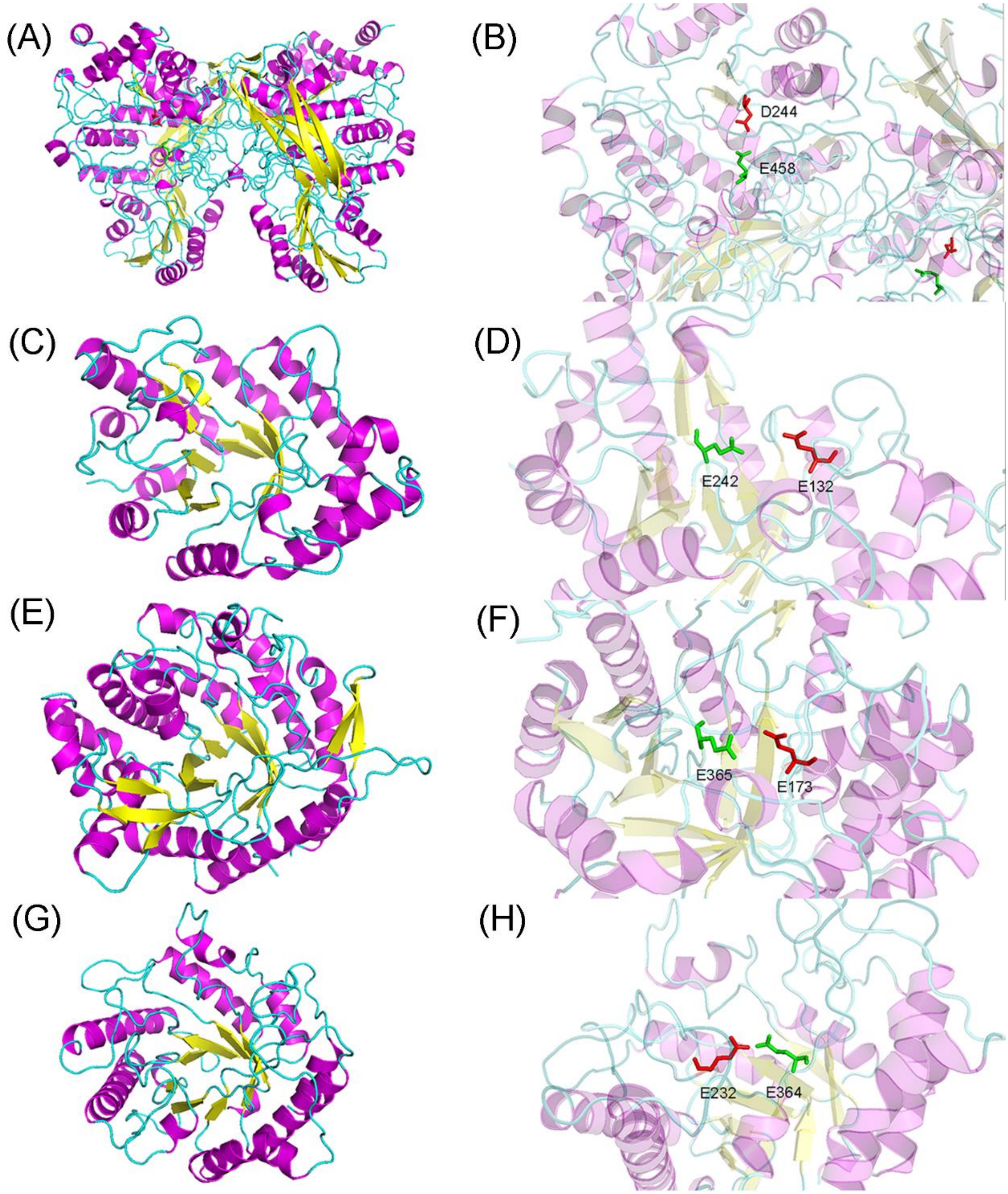

Supplementary Figure 4. Structural models and active sites of Xyl522 (A and B), Xyn526 (C and D), Bgl8520 (E and F) and Cel1753 (G and H)

(A), (C), (E) and (G) represent the overall structures of four enzymes with colored secondary structure. The helix, sheet and loop are colored magenta, yellow and cyan, respectively. (B),

(D), (F) and (H) show the active sites of four enzymes. The catalytic nucleophile and catalytic proton donor of Xyn526, Bgl8520 and Cel1753 are glutamic acid (Glu), and for Xyl522 are aspartic acid and glutamic acid. Catalytic nucleophile and catalytic proton donor are colored red and green, respectively. The templates used for Xyl522, Xyn526, Bgl8520, and Cel1753 modeling were 3u48.1.A, 4k68.1.A, 1w3j.1.A, and 1edg.1.A, respectively.
