# Supplementary material for: Synergistic function of four novel thermostable glycoside hydrolases from a long-term enriched thermophilic methanogenic digester
Source: Front Microbiol. 2015 May 22;6:509. doi: 10.3389/fmicb.2015.00509 (PMC4441150; doi:10.3389/fmicb.2015.00509)
Supplement: Supplementary file 11 [file Image7.PDF]

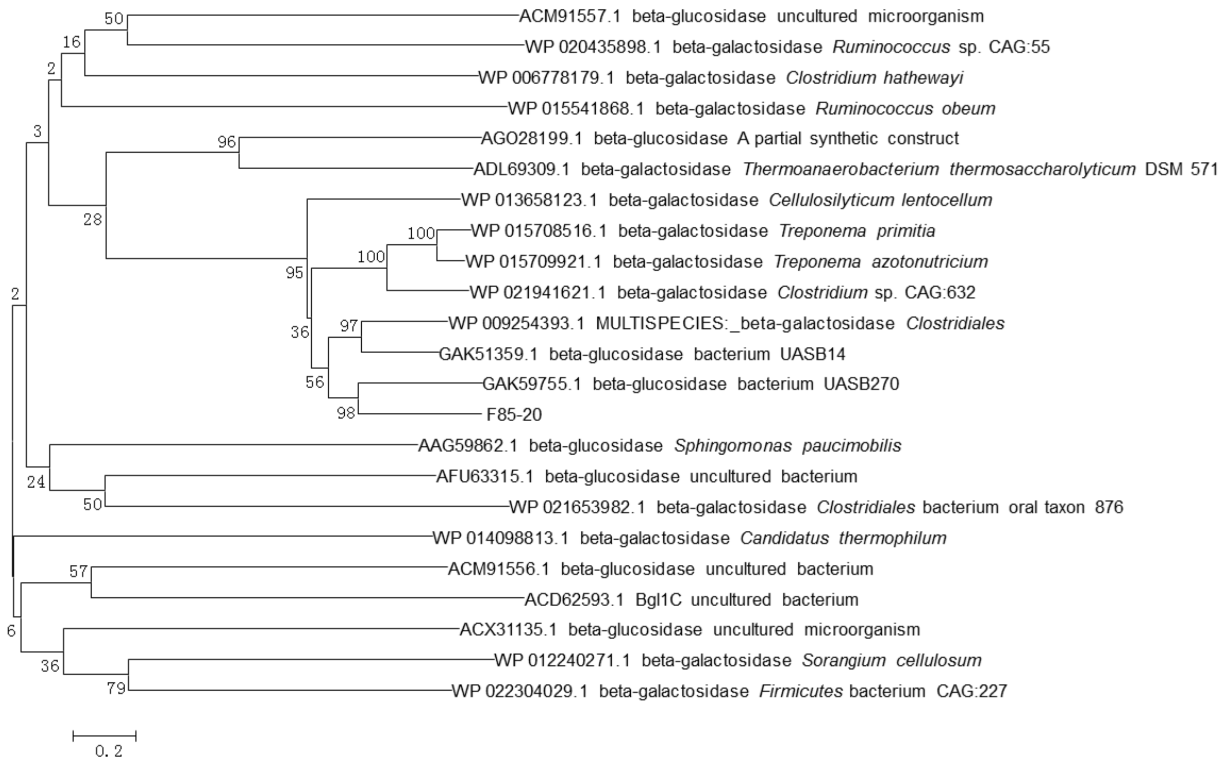

Supplementary Figure 7. Phylogenetic relationship of Bgl8520 (F85-20)

Sequence aliginment was performed by ClustalW and the phylogenetic trees were construcuted by MEGA 5.1 using the neighbor-joining algorithm based on homologous amino acid sequences.
